# Osteoporosis and dermatoporosis: a review on the role of vitamin D

**DOI:** 10.3389/fendo.2023.1231580

**Published:** 2023-08-24

**Authors:** Fiammetta Romano, Domenico Serpico, Mariateresa Cantelli, Antonella Di Sarno, Carmine Dalia, Rossana Arianna, Mariarosaria Lavorgna, Annamaria Colao, Carolina Di Somma

**Affiliations:** ^1^ Endocrinology Diabetology and Andrology Unit, Department of Clinical Medicine and Surgery, University of Naples “Federico II”, Naples, Italy; ^2^ Dermatology Unit, Department of Clinical Medicine and Surgery, University of Naples “Federico II”, Naples, Italy; ^3^ Internal Medicine S. Maria Della Pietà Hospital Nola, Nola, Italy; ^4^ UNESCO Chair “Education for Health and Sustainable Development”, University of Naples “Federico II”, Naples, Italy

**Keywords:** dermatoporosis, osteoporosis, skin, skin aging, aging, glucocorticoids, vitamin D

## Abstract

Osteoporosis (OP) and Dermatoporosis (DP) are expressions of the aging process at the skin and bone levels, respectively. Both conditions are associated with increased morbidity for elderly people, and this requires necessary interventions. They share many common risk factors; among these, vitamin D (VD) deficiency appears to have a role. VD is involved in either disease with many mechanisms, among which immunomodulation. VD deficiency has been linked to OP because it inhibits the body’s capacity to absorb calcium and maintain optimal bone health. Available evidence suggests that proper vitaminosis D also appears to be vital in preventing skin age-related issues. DP is often seen in elderly individuals, particularly those with long-term sun exposure and a history of chronic sun damage. VD deficiency can be linked to DP, since its involvement in collagen production, epidermal barrier function, inflammation regulation, wound healing, and sun protection. Aim of this review is to summarize the most updated existing evidence on the role of VD in the development of fragility syndromes such as DP and OP and the possible benefits of VD supplementation as a simple and harmful weapon against aging.

## 1 Introduction

The most frequent metabolic bone disorder is osteoporosis (OP). It is distinguished by low bone mineral density (BMD) and decreased bone strength, which increases the risk of fragility fractures. OP is the leading cause of bone fractures in the elderly, making it a substantial public health issue with a large impact on health systems (1). Kaya and Saurat created the term dermatoporosis (DP) to describe an excessive cutaneous fragility induced by increasing loss of the skin’s protective mechanical function with age. The word DP derives its name from the similarities to OP-induced bone fragility (2): just as in OP we witness a decline in the mass and quality of skeletal tissue, so in DP the structural elements of the skin barrier are lost (3). OP and DP share many common risk factors, such as aging, sex and corticosteroid use. Among these, a lack of vitamin D (VD) could have a role. The VD impact on skeletal health is well known and it is universally recognised the importance of its supplementation in elderly patients suffering from bone loss (4), however VD actions are involved in many tissues and skin is one of them. In fact, when there is adequate UV-B irradiation, this organ is capable of producing itself the active form of VD which is 1,25(OH)2D. The latter 1,25(OH)2D has crucial roles such as the control of epidermal barrier integrity (5). Recent findings indicate that VD regulates aging in various tissues, including the skin (6). Our review sought to investigate the links between OP and DP, as well as the available information on the function of VD in the onset of these conditions and the possible therapeutic use of VD supplements.

## 2 Osteoporosis in elderly

### 2.1 Definition and epidemiology

OP is a systemic skeletal illness marked by low bone mass, micro-architectural degeneration of bone tissue, bone fragility, and an increase in fracture risk (especially of vertebrae, femur, humerus, wrist and ankle bones) due to even minimal trauma (7). The epidemiological impact is very high: nowadays it is believed that in Italy around 5 million elderly people are affected by OP and a greater increase in its incidence is expected in the next future, since the proportion of the Italian community of over 65 years is going to rise by 25% in the next 20 years. Osteoporotic fractures raise the relative risk of mortality, particularly for femur fractures: it is 5-8 times greater in the first 3 months after the occurrence, decreases in the next 2 years, but stays high even after a 10-year follow-up. Furthermore, 50% of women with hip fractures had a significant loss in self-sufficiency which involves long-term institutionalization in about 20% of cases. The economic burden of such a widespread pathology is therefore very high (8, 9). The World Health Organization operationally defines OP as the presence of a bone mineral density (BMD) of 2.5 SDs under the average of young white adult women (1). OP is classed as ‘primary’ when it is not caused by medical conditions, and secondary’ when it arises as a result of particular, well-defined clinical diseases or drug use (10).

### 2.2 Elderly osteoporosis: pathogenesis

Under physiological conditions, skeletal homeostasis is guaranteed by an appropriate ratio between formation and resorption of bone tissue. Skeletal homeostasis is maintained under physiological settings by a balance of bone production and bone resorption. This adjustment is altered in pathological circumstances in favor of osteoclast-mediated bone resorption (5).

This bone regeneration cycle decoupling is exactly what happens in elderly people: the decreased osteoblast activity determine a longer time required to fill resorption cavities and there is a low-grade systemic inflammation, especially involving pro-inflammatory cytokines [tumor necrosis factor-alpha (TNF-alpha), IL-1, and IL-6)] which determine an increase in the amount and functioning of osteoclasts. As a result, in older persons there is an overall decline in bone with time. A negative calcium balance resulting from decreased dietary intake, reduced absorption and the compromise of kidney function, reduces the activation of VD and the calcium absorption from the gut (11). Osteoclasts indeed must resorb calcium in order to fill this void (4). Estrogen deficiency is of course another critical factor responsible for the increased bone resorption both in men and women. For either sex, bone loss occurs right after attaining maximal bone mass; nevertheless, this process accelerates after menopause in women and after the age of 70 in men (12). Estrogens are well known for regulating the synthesis of bone. They have a bone-protective role by limiting bone resorption and sustaining bone formation (13). As a result, estrogen deprivation causes OP, which is associated with an increase in bone resorption due to a boost in the number and activity of osteoclasts, as well as osteocyte death. A growing body of information suggests that OP related with estrogen loss of estrogen is also due to the increase in oxidative stress and changes in immune system homeostasis and inflammatory pathways, which are accentuated by the aging process. Specific T-cell subsets, such as T helper cells, can be activated, supporting the production of IL-17, Receptor activator of nuclear factor-Kappa B Ligand (RANKL), IL-1, TNF, and IL-6. These factors are able to stimulate osteoclast maturation and activity by preventing the differentiation of osteoblast, increasing apoptotic osteocytes, and raising RANKL expression and the RANKL/Osteoprotegerin (OPG)-ratio (14).

### 2.3 Elderly osteoporosis: clinical features

OP is asymptomatic for the majority of its clinical history; approximately one-third of fracture occurrences are silent, while the remainder present in pain. Osteoporotic fractures are fragility fractures, meaning they occur spontaneously or as a result of little trauma. In order of frequency, the most commonly affected sites are vertebra, femur, major non-vertebral/non-femoral fractures (pelvis, radial tip, proximal tibia, humerus, 3 or more ribs, etc.), minor fractures followed by pain. Vertebral compression fractures (VCFs) are the hallmark clinical presentation of OP. Unlike major posttraumatic VCFs, which are invariably symptomatic, those caused by moderate trauma are frequently misunderstood and thus go undiagnosed. They are typically asymptomatic or present with symptoms such as back or low back pain that responds to analgesic therapy (15, 16). They primarily impact the dorsolumbar junction (T12-L1), followed by the mid-dorsal tract (T7-T8) and other locations (17). The Genant classification, which takes into account the level of vertebral body involvement by a semi-quantitative evaluation of its deformity, is used to categorize the severity of vertebral fractures (18). The reduction in the patient’s height, which can be partly attributed to antalgic posture and partially to the accentuation of the thoracic kyphosis, is the evident and immediately observable result of vertebral collapse. The latter causes a number of issues, including sleep disturbances caused by adopting analgesic positions, limitations on daily activities, respiratory (modest reduction of respiratory volumes due to reduction of rib cage support) and gastrointestinal (early satiety due to abdominal distension) disturbances. Neurological deficits occur rarely, even when they cause compression of the spinal cord (by sliding) (19). Hip fracture is a severe injury that necessitates hospitalization and immediate medical surgery (20). Such fractures account for a small percentage of fractures caused by OP (about 15%), but they have a greater impact on health expenditure (21) because they are associated with a higher rate of morbidity and mortality, particularly in the first three months after the fracture; numerous complications are in fact associated with this event: embolism, pulmonary disease, infections, sepsis, heart attack, and cardio-pulmonary problems in general (22). Hip fracture risk increases with age, and so does mortality (by 5-8 times): this connects with both BMD decline and an increase in the chance of falling (which accounts for around 90% of fractures in the elderly) (21). Colles fracture, or fractures of the distal epiphysis of the radius, are more common in persons with a higher performance level because they are more active and hence at a higher risk of falling. Its prevalence rises gradually after menopause, then levels out at the age of 65. In contrast, wrist fractures are uncommon in men, regardless of age (M:F 1:6-1:10 at 65 years) (21). This disparity is related to the male skeleton’s bigger cortex and lower endocortical resorption (23). Other types of fractures (humerus, pelvis, proximal radius, or distal femur) are more uncommon. Although the association with age is indisputable due to the loss in BMD, it is relatively weak for rib fractures and more meaningful for pelvic fractures (21).

## 3 Dermatoporosis

### 3.1 Definition and epidemiology

Skin aging is not just an aesthetic problem. In fact, its effects also weaken the skin on a functional level, reducing the crucial protective properties of the skin. Since this growing knowledge in recent years, it has been necessary to coniate a term as DP that could focus the attention of clinicians on the urgency of preventing and treating this condition as well as OP.

DP is a clinical entity that includes the whole broad spectrum of skin alterations due to aging, including complications that can cause severe morbidity for the affected elderly person, taking the form of a real chronic skin frailty syndrome (2). People with dermatoporosis have particularly thin and fragile skin. This results in a poor tolerance to friction and shear forces, and consequent susceptibility to skin tears and more or less serious hematomas. DP also tends to delay the healing of wounds once they have formed. Additionally, skin failure leads to loss of temperature regulation combined with the incapacity of keeping the core body temperature. In more severe cases, it may result in percutaneous loss of fluid with electrolytes and protein, as well as an increased susceptibility to infection (24). For this reason, severe DP is a cause of death in intensive care units (25). Similarly to OP, DP is classified as ‘primary” and ‘secondary’. Primary DP is caused by chronological aging along with long-term, unprotected sun exposure. Although data on genes associated in DP pathogenesis are limited, genetic variables are known to play a substantial role in the regulation and loss of extracellular matrix (ECM) components, in the viscoelastic characteristics of the skin, and hence could be involved in DP susceptibility. Chronic use of topical and systemic corticosteroids causes the secondary type. These iatrogenic forms may appear earlier and be more severe in people predisposed to primary DP. Corticosteroids are known to affect the expression of genes encoding collagens I, III, IV, V, decorin, elastin, metalloproteinases (MMPs) 1, 2, 3, tenascin, and MMP 1 and 2 tissue inhibitors (2).

Data on the prevalence of the disease are currently scarce, however they show the high frequency among the elderly and especially in women. The frequency of dermatoporosis is 32%, based on a study of 202 hospitalized participants aged 60 to 80 years. DP was found in approximately 22% of females and 38% of males (26). Another French study evaluated 533 people over the age of 65 who went to see a dermatologist, resulting in an overall prevalence of dermatoporosis of 37.5%, which was prevalent in women (27). Finally, in a prospective analysis of 176 patients aged 60 years or more, Kluger et al. discovered DP in 30.7% of patients, mostly in the upper extremities (94%) (28).

### 3.2 Dermatoporosis: pathogenesis

There are many factors implicated in the development of DP. First of all, the decline with aging in dermal hyaluronic acid (HA). In the elderly there is a thinning of the skin, which loses its resistance to mechanical forces. The firmness of “healthy” skin is provided by the ECM, whose major constituent is HA, a non-sulfated glycosaminoglycan that is mostly produced by fibroblasts. HA is a very hydrophilic material that decreases friction between collagen fibers and provides shear force resistance. Yet, as people age, fibroblasts lose their ability to make HA; as a result, the ECM loses volume and consequently is protective mechanical function, determining skin laceration from minimal trauma (2). The lack of interaction between HA and the cell surface receptor CD44, which normally increases keratinocyte proliferation, is a second mechanism hypothesized. In mice models, the selective suppression of keratinocyte CD44 determines skin atrophy (29). This observation has served as the foundation for DP research: it has been shown that dermatoporotic skin has lower CD44 levels than “healthy” skin from young persons (30). Expression of CD44 is also decreased by UVA and UVB exposure (31) and topical corticosteroid application (30–32). Corticosteroids can also cause dermatoporotic alterations by modifying collagen I, collagen III, collagen IV, and matrix MMP gene expression (33). With age, the overexpression of MMP 1, 2, and 3 and the downregulation of the tissue inhibitor of MMP 1 leads to the degradation of collagen and elastin in the dermis (34). The malfunction of the hyalurosome, a multimeric macromolecule complex comprising of components involved in HA metabolism and cell signaling in keratinocytes such as CD44, heparin binding epidermal growth factor, and its receptor erbB, appears to be the most critical aspect of DP (35).

### 3.3 Dermatoporosis: clinical features

Clinical manifestation is variable and includes morphological and functional alteration of the skin. Skin atrophy, senile purpura, and stellate pseudoscars are morphological signs of skin fragility. Sun-exposed areas of skin atrophy include the pretibial zones, the back of the forearms, the dorsum of the hands, the presternal area, and the scalp. Dermatoporotic skin is clinically very thin and transparent, with many wrinkles, senile purpura, and pseudoscars as compared to younger skin. There is a significant reduction in skin thickness, demonstrated by ultrasound (1.4-1.5 mm “healthy” skin thickness vs 0.7-0.8 mm in dermatoporotic skin). The dermis, which contains the majority of the subcutaneous fat, shows substantial elastosis, while the epidermis displays linearization with lack of rete ridges (2). Senile purpura, also known as Bateman purpura, is a benign superficial hemorrhagic lesion caused by repeated spontaneous or minor trauma. Histologically, it is distinguished by erythrocyte extravasation and enhanced vascular fragility as a result of connective tissue injury and atrophy in the dermis. These dermal bleedings are not associated with any coagulation disorders (3). Senile purpura is very common in up to 10% of the elderly population between the ages of 70 and 90 and in 90% of cases it is associated with pseudoscars. As these purpuric plaques fade, a dark brownish pigmentation that resembles hemosiderin pigment is left behind (36). Vitamin C, whose protective role on blood vessels is well known, is often deficient in elderly people with dermatoporosis, leading to dermal hematomas; so acid ascorbic replacement therapy should be considered in this pattern (2). Stellate Pseudoscar is a superficial lesion of the skin with a star shape, deriving from spontaneous dermal laceration caused by minimal trauma. These lesions have a hypocellular dermal zone in a background of fibrosis and elastotic collagen fibers covered by an atrophic epidermis. As the senile purpura, Stellate Pseudoscar appears on the backs of elderly people’s hands and forearms and they show as pale lesions on medical inspection. Pseudoscars are classified into three kinds based on their shape: star-shaped, linear, and plaque (36).

Functional manifestations of DP are skin lacerations, delayed wound healing, and subcutaneous bleeding with the development of deep dissecting hematomas (DHH) and in the most serious cases even large areas of necrosis (3). DHH is the most serious DP complication. It occurs as a result of extensive bleeding between subcutaneous fat and muscle fascia following a small injury (37). DHH typically occurs in elderly patients with dermatoporosis who underwent anticoagulation therapy or topical or systemic corticosteroid therapy (33). Histologically, there is exposure of deep skin vessels to the skin surface in the context of significant skin thinning. Unlike the other clinical signs of DP, DHH occurs mostly in the lower limbs of elderly adults with severe DP (M/F ratio: 1/5) (38). Necrosis is caused by large hematomas that cut off the blood flow to the skin. As soon as possible to prevent severe skin damage in this situation, the bleeding area and necrotic tissue should be surgically removed. It may be essential to make large and deep incisions that reach the muscular tissue, determining a significant loss of skin surface (39). The exact mechanism causing the delay in wound healing in elderly is still unclear. Tissue deterioration is caused by a reduction in the ability of keratinocytes and fibroblasts to proliferate, a surplus of matrix MMPs, which delay the production of renewed ECM (40).

## 4 Osteoporosis and dermatoporosis: common risk factors

**Figure 1:**
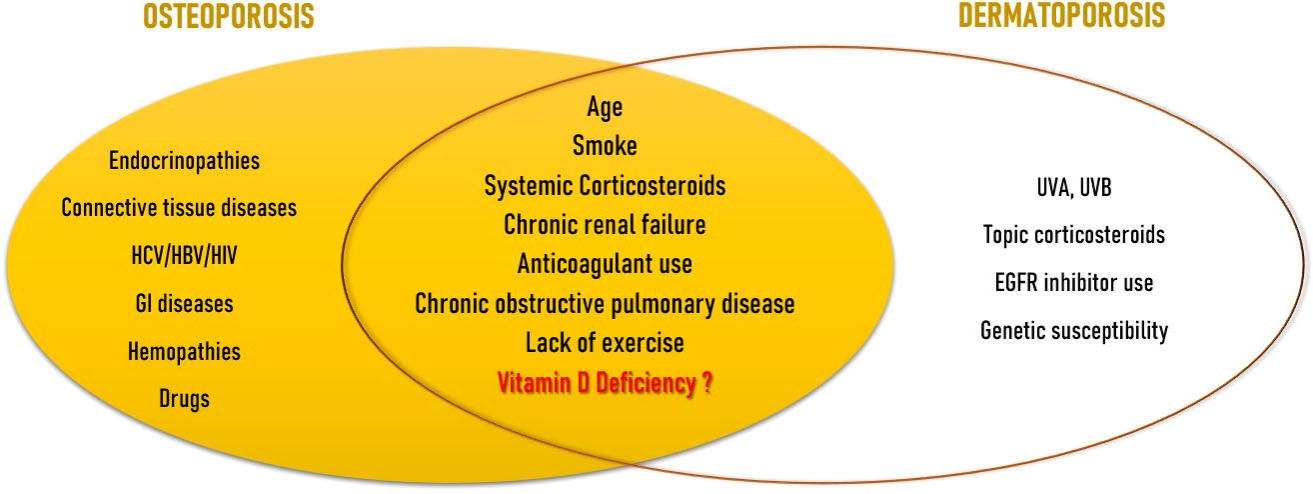
Dermatoporosis and Osteoporosis: common risk factors.

OP and DP share many common risk factors (**Figure 1**). The major risk factor for both illnesses is advancing age. Aging exponentially raises the likelihood of osteoporotic fractures, only partially due to the observed reduction in BMD, but also due to many other factors accompanying aging, namely alterations in bone quality, a rise in the number of falls, and a slowing of defensive reactions. In fact, fracture risk can be higher in elderly than young patients even if in presence of similar BMD (8). Clinical signs of DP appear from the age of 60, and are more noticeable at between 70 and 90 years of age (41). Observational studies on subjects attending dermatology units aged 60 or older (28, 42) or on geriatric rehabilitation patients (43) showed that age was an independent risk factor for the development of DP. Another well recognised risk factor is smoke. Tobacco use is an independent risk factor for both vertebral and limb fractures (8). Current smokers exhibit a weaker inverse relationship between PTH and serum VD levels compared to nonsmokers. They also lose more BMD over time, particularly at the femoral site, and are more likely to experience fragility fractures (44). Smoking was found to be significantly associated also with DP (43). Use of systemic corticosteroids represent the main form of secondary OP and is distinguished by a qualitative change in the skeleton as well as in the macro and bone microarchitecture (45). As seen above, corticosteroids are involved in the regulation of the expression of genes that encode collagens I, III, IV, V, decorin, elastin, MMPs 1, 2, 3, tenascin and tissue inhibitors of MMPs 1 and 2 (33). Kluger et al. discovered that use of both topical and systemic corticosteroids was strongly linked with DP in a Finnish observational research (28). Chronic renal failure causes the condition named as “Chronic Kidney Disease-Mineral and Bone Disorder” (CKD-MBD) which is characterized by a group of alterations in calcium-phosphorus metabolism, changes in hormones involved in bone homeostasis such as parathyroid hormone (PTH), VD and fibroblast growth factor-23 (FGF-23), anomalies in bone turnover and mineralization, and vascular calcification. The risk of fracture and vascular disease increases as a result of all these factors (46). Dermatoporosis and chronic renal failure have also been linked in a strong, age-independent way (28, 42). Chronic renal failure was revealed to be the only age-independent factor that significantly increased the incidence of DP more than five times by multivariate analysis in the cross-sectional observational investigation by Mengeaud et al. (26). Hyperpigmentation and haematomas, which are seen in most advanced stages of DP, are frequently documented in patients with end‐stage renal disease; however, there is no convincing understanding for the link between severe chronic renal disease and DP. Anticoagulant use has shown the most important association for the development of DP in observational studies (27, 42, 43); anticoagulant use and chronic renal disease seems to act as additional cofactors (28). The prevalence of OP in individuals with chronic obstructive pulmonary disease (COPD) ranges from 9 to 69% (47). In this setting, OP-related fractures are associated with several adverse health outcomes, including an increase in hospitalization and mortality rates, in lung function, and poor quality of life (48). In a cross-sectional analysis of individuals 65 and older, Reszke et al. found that those with COPD were at higher risk to demonstrate senile purpura, although it could be a consequence of systemic corticosteroid use (49). Lack of exercise is seen as a moderate risk factor for OP and fragility fractures (8), as well as for DP (50). Since the observation that DP and OP share many common risk factors, Villeneuve et al. proposed that DP could be a sign of underlying bone fragility. In their prospective, observational, cross-sectional, multicenter study on patients of 50 years or older, they found a link between DP and a history of significant osteoporotic fracture, regardless of age or gender (51). Not much research has been done on the role of VD insufficiency as a risk factor that both diseases share. However, there is evidence that VD is involved in both skin (52) and bone health (53).

## 5 Vitamin D

### 5.1 Metabolism

Besides being a lipid-soluble vitamin, VD is a steroid hormone. Humans obtain VD from either sunlight exposure to UVB rays or minimally from the introduction with plant or animal foods, dietary foods and supplements, as VD2 (or ergocalciferol) or VD3 (or cholecalciferol) (54). VD is produced in the skin via 7-dehydrocholesterol (7-DHC) present in keratinocytes; UVB rays mediate a photochemical reaction that converts 7-DHC in vitamin D3 (55). The latter is transported to the liver binded to a type of alpha-globulin called Vitamin D Binding Protein (VDPB), where it undergoes the first hydroxylation and is released in the form of 25(OH)D3. Finally, VD3 is activated with a second hydroxylation at the kidney level. The proximal tubule is the primary site of action for this mechanism, and it is vulnerable to both negative and positive feedback processes mediated by 1,25(OH)2D3, phosphorus, calcium, and FGF-23 (56). The 1,25(OH)2D3 acts through activation of the nuclear receptor of VD (VDR), a transcription factor and part of the steroid receptor family, with which VD has a high affinity. Although the primary function of VD, which is the control of calcium-phosphorus metabolism and the balance of bone mineral reserves, is well understood (57, 58), there is also ample evidence of its numerous other activities in extra-skeletal tissues (59, 60). In reality, the VDR is expressed everywhere (61), and VD is essential for the immune system’s physiology (62), for controlling the activity of other hormones like IGF-1 (63), in the prevention of many types of neoplasms (64), in the maintenance of a solid skeletal muscle (65), participates in carbohydrate metabolism (66), of the cardiovascular (67) and reproductive systems (68), of the neuro cognitive (69) and cell proliferation (70, 71). As we will see in more detail later, it is relevant also in the constitution of the skin (72). VD seems to act either directly on organs such as bone and skin, and indirectly through influencing the immune system and, in turn, inflammatory processes, which is a major factor in the development of many diseases like OP and skin aging. All immune system cells have VDR, and antigen-presenting cells (APC) can create 1,25(OH)2D3 in response to immunological stimuli by using the same enzyme that is produced in the kidney (62).

Both innate and acquired immunity are affected by 1,25(OH)2D3. VD and its metabolites influence innate immunity by promoting macrophage development and activation, which results in the production of defensins including cathelicidin and 2-defensin (73). Mice on a diet lacking in vitamin D produced less IL-6, TNF-, and IL-1, and their antibacterial activity was weak (74). The primary inhibitory effects of VD on acquired immunity result in a phenotypic shift in T cells from an effector phenotype, which is involved in autoimmune disorders, to a regulating and protecting one (75).

### 5.2 Vitamin D deficiency: definition and epidemiology

The interpretation of serum 25(OH)D has to take into account many factors, as levels can vary widely in different life periods, based on degree of exposure to sunshine (period of the year, latitude), phototype, and nutritional status (76). There is also a large variability in its dosage between different laboratories. In fact, there is still no unanimous consensus among scientific societies for the definition of the deficiency of VD, except for the condition of serious deficiency represented by values ​​of 25(OH)D <10 ng/mL which are linked with higher risk of rickets and osteomalacia. Since the observation that in the general population there is a relationship between values of serum 25(OH)D < 20 ng/mL and increased risk of fracture (77), the Italian Society for Osteoporosis, Mineral Metabolism and Bone Diseases (SIOMMMS) suggests to consider these cutoffs: “deficient” means a 25(OH)D level of 10 ng/mL; “insufficient” means a level of 20 ng/mL; and “optimal” means a level of 20-50 ng/mL (76). In patients with OP, especially those who necessitate a treatment with OP drugs and subjects at risk of hypovitaminosis D, an “optimal” level of at least 30 ng/mL is instead indicated. This value is related with a considerable reduction in the incidence of hip fractures in institutionalized women and a 4.5 times better response in bisphosphonate-treated patients (78). Globally, there are many people who have mild or severe VD deficiencies. Around 7% of the world’s population has severe VD deficit (serum 25(OH)D concentrations below 25/30 nmol/l (10/12 ng/ml)), while 37% has mild VD deficiency (serum 25(OH)D concentrations below 50 nmol/l (20 ng/ml)) (79).

### 5.3 Vitamin D and bone health

#### 5.3.1 Effects on bone homeostasis

VD is required to increase the active intestinal absorption of calcium by 30-80% which, later, becomes available for multiple physiological processes and for the mineralization of the skeleton. Additionally, it promotes calcium reabsorption in the kidney’s distal tubule. By encouraging osteoblast development and regulation as well as the generation of proteins including collagen, alkaline phosphatase, osteocalcin, and RANKL, 1.25 (OH)2D3 also has direct effects on bone. It controls both bone formation and resorption (80). Intestinal calcium and phosphate absorption significantly decreases when serum 25(OH)D is less than 30 ng/mL. The blood ionized calcium concentration is lowered as a result, which leads to secondary hyperparathyroidism. Increased osteoclast activity is caused by preosteoclast differentiation into mature osteoclasts, which is induced by elevated PTH levels. Increased bone resorption, loss of bone matrix, and resultant reduced bone mass are the outcomes of this. Due to the PTH-induced increase in osteoclast activity and quantity, VD-deficient osteons exhibit broader Haversian canals and greater lacunae. This increases porosity. Additionally, osteoid mineralization is defective when compared to that of normal bone (53). Clinical manifestations of VD deficiency reflect all these functions. Severe deficiency leads to an insufficient calcium-phosphate product: the result is broadly deficient osteoid mineralizations. Rickets, which manifests as poor mineralization throughout the developing skeleton, and osteomalacia, which results from impaired skeletal mineralization after the fusion of epiphyseal plates in adults, are the clinical consequences (81). 25(OH)D levels in rickets and osteomalacia patients typically fall below 15 ng/mL (82).

#### 5.3.2 Vitamin D and osteoporosis

Less severe degrees of deficiency may also produce skeletal disease. In fact, long-standing VD deficiency/insufficiency (serum 25(OH)D level lower than 30 ng/mL) is considered a risk factor of OP because of the mechanisms that increase resorption seen above (53). Epidemiologic studies show that VD deficiency is associated with lower BMD and fractures. In the Longitudinal Aging Study Amsterdam (83) 25(OH)D levels and BMD of lumbar spine and hip of 1319 subjects (643 men and 676 women) between the ages of 65 and 88 yr were correlated. It was found a threshold around the serum 25(OH)D level of 50 nmol/liter for the relationship between serum 25(OH)D and BMD of total hip and femoral trochanter. Kuchuk and colleagues also found an association between VD deficiency and fractures. Serum 25(OH)D levels below or equal to 30 nmol/L were associated with an increased fracture risk in persons aged 65 to 75 years (83). Also longer follow-up studies show a similar increase in fracture among subjects with the lowest VD status (84). Besides epidemiologic observations, contradictory findings have been obtained from the numerous intervention trials conducted in elderly individuals to determine if VD supplementation alone or with calcium can reduce the risk of fractures (85). VD’s anti-fracture impact has mostly only been observed for femoral fractures and non-vertebral fractures, not vertebral ones. It also appears to be mediated by the reported decline in the risk of falling (86). Data on elderly people indicate very clearly that the skeletal benefits of the VD supplementation are seen in those who are severely VD and not if they have mild or no VD deficit (87, 88). In the New Zealand Vitamin D Assessment (ViDA) study of older community-resident men and women treated with monthly dosing of 100,000 IU VD for 2 years, clinically significant reductions in bone loss at the spine and femoral neck, were found only in participants with a baseline serum 25(OH)D < 30 nmol/L (87). Subsequently, in the Aberdeen study (88), authors aimed to verify if the baseline 25(OH)D threshold of <30 nmol/L was confirmed. 305 postmenopausal were randomized to receive either VD 400 IU/day or 1000 IU/day, or placebo over 1 year. Results of a *post-hoc* analysis confirmed the usefulness in terms of increasing BMD of the VD supplementation only in the group with a baseline level of 25(OH)D ≤ 30 nmol/L (88). Benefits of VD supplementation seem to be enhanced when combined with calcium, a non surprising observation since it is well known that elderly people are often at high risk of contemporary VD and calcium deficiency (89). In the 2019 meta-analysis by Yao and colleagues, VD reduced the risk of any fracture by 6% and of hip fracture by 16% but only when supplementation consisted also in calcium (90). In any case, an adequate intake of calcium and VD is the prerequisite for any specific drug treatment since calcium and/or VD shortage is one of the most frequent reasons for failure or a poor response to osteoporosis medication (91, 92). This could obviously enhance the risk of future further fracture (93). As discussed earlier, OP may have also an inflammatory etiology (14); it is possible, given the immunoregulatory effects of VD (62), that its benefits on fracture risk may at least be partially mediated, at least in part, by an influence of VD on cytokine concentration. In the research conducted by Inanir et al., 70 post-menopausal women diagnosed as osteoporotic were randomized to receive calcium and calcitriol or calcium alone. At baseline and six months into the course of treatment, measurements of BMD and serum levels of IL-1, IL-6, and tumor necrosis factor-alpha (TNF-alpha) were made. According to study findings, taking 20 IU of calcitriol every day for six months enhanced BMD and lowered IL-1 and TNF-a concentrations (94).

### 5.4 Vitamin D and skin

#### 5.4.1 Effects on skin

Skin and VD have a special relationship: skin is in fact the only organ that can produce VD and its metabolites, also being at the same time a major target for this hormone as well (95). Keratinocytes express all enzymes of the VD metabolic pathway and can produce hormonal 1,25(OH)2D3 when exposed to enough UV-B irradiation. Thusly produced 1,25(OH)2D3 acts in many ways at the skin level, with three most important actions: regulation of keratinocyte proliferation and differentiation, control of epidermal barrier integrity (5), and modulation of the immune skin system (96). Different receptors in the skin have varied affinities for VD and its CYP11A1-derived hydroxyderivatives, which allows them to exert a variety of partially overlapping actions. The binding to the nuclear VDR plays a significant role in mediating the biological consequences (97). A non-genomic, membrane-associated approach based on a different ligand-binding site (98) or the action on the 1,25D3-MARRS receptor (99) can also be used by the activated VDR to cause rapid response signaling. Retinoic acid-related orphan receptors (ROR) α and γ, which are expressed in the skin, are two additional nuclear receptors that VD metabolites can use to control some skin activities (100). Last but not least, the traditional 1,25(OH)2D3 and CYP11A1 derivatives can bind to the liver X receptors (LXR) and aryl hydrocarbon receptors (AhR) and operate as agonists (101, 102). Through intracrine, autocrine, and paracrine effects, 1.25(OH)2D3 produced in keratinocytes controls their own development, differentiation, and death (103). Specifically, VD causes in vitro keratinocyte growth to be stimulated at low doses and inhibited at higher concentrations (108 M) (104). Additionally, it preserves the integrity of the epidermal barrier by promoting the synthesis of ceramides, key players in the control of the skin’s water-holding capacity and homeostasis (105). In a feedback loop, ceramide boosts the pro-differentiating effect of calcitriol on keratinocytes when VD stimulates the neutral Mg2+-dependent sphingomyelinase (106). Physiological levels of calcitriol inhibit the effects of pro-apoptotic ceramides, UV radiation, and TNF-α, whilst pharmaceutical doses cause keratinocytes and other epidermal cells to undergo apoptosis (107).

As previously reported, the presence of VDRs in almost all immune cells suggests that they are one of vitamin D’s primary targets, and various immunological indicators are controlled by VDRs action (108). This happens also in the skin, where VD and its metabolites exert multiple actions on T-cells, dendritic cells, keratinocytes and myeloid cells (109, 110).

Overall, VD has an immunomodulatory effect on T cells (52). VD inhibits proinflammatory Th1/Th17/Th9-Lym T-cells activation (111), as well as the generation of inflammatory cytokines (interferon gamma, TNF-α, IL-2, IL-17/21) (111–113), while increasing the levels of anti-inflammatory IL-10 and IL-4 (114, 115). As a consequence, VD increases the production of CD25+/CD4+ regulatory T cells, shifting the Th1 inflammatory response towards the more tolerogenic Th2 response (116). Following antigen stimulation, VD directly controls the expression of the antimicrobial peptide (AMP) gene in innate immune cells, promoting tolerance and inhibiting immunity (117, 118). VD causes the surface of T-cells to express the CCR10 receptor, which enables them to migrate from dermal blood arteries to epidermal keratinocytes (119).

#### 5.4.2 Vitamin D and dermatoporosis

Several studies on VD receptor mutant mice have put the basis for the knowledge of VD relevance in controlling aging in skin and many other tissues, as these mice developed typical phenotypic traits of premature aging such as skin and overall body atrophy as well as OP. By normalizing mineral VD, these phenotypic traits can be reversed (6, 120–122). Surprisingly, the aging phenotypes of mice with hypovitaminosis D (VDR−/− and CYP27B1−/− mice) are strikingly comparable to those of mice with hypervitaminosis D (including FGF-23−/− and Klotho−/− mice) (6, 121). Keisala et al.’s study used VDR “Tokyo” knockout (KO) mice to examine growth, skin and cerebellar morphology, as well as general motor function. They discovered that the phenotype of old VDR KO mice was comparable to old hypervitaminosis D3 mouse models, indicating that VDR genetic ablation accelerates early mouse aging (121). Therefore, vitamin D deficiencies, both mild and severe, may speed up aging. According to VD status, aging actually appears to follow a U-shaped curve, making adequate levels of VD important regulators of the physiological aging process and essential for avoiding premature aging (120).

Aside from its potential application in the treatment of skin aging for aesthetic purposes, there is evidence that VD can also play a role in the prevention and management of DP and its severe repercussions. Here we summarize some key ways in which VD influences skin health and its potential impact on DP.

First of all, in DP there is an impairment of the collagen component of the skin (2). As discussed above, collagen, a protein responsible for skin strength and elasticity (123), tends to decrease with age, contributing to the thinning and fragility of the skin (2). VD has been found to stimulate collagen synthesis, promoting skin thickness and resilience (124). By enhancing collagen production, VD may help counteract the effects of DP and support overall skin health. A second aspect is the regulation of epidermal barrier function. The latter is vital for maintaining skin hydration and protection against external stressors (125). VD affects the expression of genes that contribute to skin barrier development and maintenance. It helps strengthen the protective layer of the skin, reducing water loss and improving the skin’s ability to defend against environmental factors that contribute to DP (126). Anti-inflammatory effects are of course involved. Chronic inflammation is a key contributor to skin aging and DP. An immunological change and an imbalance between pro- and anti-inflammatory mechanisms cause a chronic low-grade inflammation state known as “inflammaging” (127, 128), which is brought on by both persistent oxidative stress and chronic antigen stimulation (129, 130). With advancing age, skin immune system presents a deep remodeling, resulting in a decrease in its capacity for adaptation (131, 132). As deeply discussed before, VD possesses anti-inflammatory properties, modulating the immune response in the skin and reducing inflammation (52). By mitigating inflammation, VD may help alleviate the symptoms associated with dermatoporosis and promote healthier, more resilient skin.

Impaired wound healing is a common characteristic of dermatoporotic skin (2). VD has been demonstrated to improve wound healing by encouraging cell proliferation and migration and facilitating collagen synthesis (133). By supporting the healing process, VD may help improve the recovery time of wounds and minimize the risk of complications in dermatoporotic skin.

Finally, VD is involved in protection against harmful UV radiation. DP skin is more vulnerable to sun damage and should be shielded from excessive sun exposure (134). The idea that 1,25(OH)2D3 has a cytoprotective effect against the harmful impacts of UV and other agents, that may aid in preventing premature skin aging, is strongly supported by a number of in vitro investigations (135–138). Oral administration of high-dose vitamin D3 immediately following exposure to UVB light reversed photo-induced cutaneous injury quickly in a double-blinded, placebo-controlled interventional trial on 20 healthy adults by reducing inflammation and inducing the epidermal barrier’s repair mechanisms (139).

Only one MR trial has looked at vitamin D status and skin phenotype. Higher observed serum 25OHD concentrations were linked to perceived age, skin wrinkling, and pigmented spots, according to research by Noordam et al. on facial skin aging features in about 4500 Dutch individuals. However, according to genetic predictions, serum 25OHD was not linked to these skin characteristics. This seems to suggest that the cause of skin aging is exposure to UV-B light rather than serum 25OHD concentrations (140).

### 5.5 Vitamin D deficiency: therapy

Everyone in the aging population, regardless of bone health status, should get enough VD (together with an adequate calcium intake of 800-1000 mg/day as well) (4). The Bone Health and Osteoporosis Foundation (BHOF) advises 800 to 1000 international units (IU) of VD per day for persons over the age of 50, while the Institute of Medicine (IOM) recommends 600 IU per day till the age of 70 and 800 IU per day for adults over the age of 71. It is however very common that older individuals develop VD deficiency, which is caused mostly by being institutionalized or chronically ill with inadequate sun exposure, absorption problems, chronic renal illness. If enough and non-hazardous solar exposure is insufficient to obtain the necessary amounts of VD for skeletal occurrences (fractures and falls), oral supplements should be used. Treatment must be personalized. Cholecalciferol is the first line therapy in most patients. No one who requires supplementing will respond to a single set dose; instead, a dose between 800 and 2000 IU per day should be taken into consideration (141). The daily method of supplementing is the most physiological; however, from a pharmacological perspective, the use of similar weekly or monthly doses is recommended in order to increase adherence to therapy. In individuals who require quick vitamin D level normalization (symptomatic osteomalacia or zoledronic acid or denosumab initiators) it is recommended to use a initial loading dose of either cholecalciferol in a single dosage of 60,000 to 150,000 IU, followed by a maintenance dose, or 3,000–10,000 IU/day (mean 5,000 IU/day) for 1–2 months (142). As an alternative, it is feasible to utilize calcifediol for 20 to 30 days before moving to cholecalciferol for maintenance dose. Since its pharmacokinetics are different from the cholecalciferol’s one because it has a lower volume of distribution, calcifediol causes a 25(OH)D level increase more quickly. In obese subjects it is suggested to use either cholecalciferol at a dosage increased by approximately 30% of the usual dose or calcifediol. The latter can also be indicated in other conditions of 25-hydroxylation deficiency which are often observed in older people, such as severe liver failure, male hypogonadism or intestinal malabsorption (79). Chronic renal disease is another ailment that is typically common in aged persons. In this setting it is recommended to use cholecalciferol and to restrict the administration of active vitamin D compounds (calcitriol or synthetic analogues) to dialysis patients or those in the G4-G5 phase with severe and progressive hyperparathyroidism (143).

## 6 Conclusions

**Figure 2:**
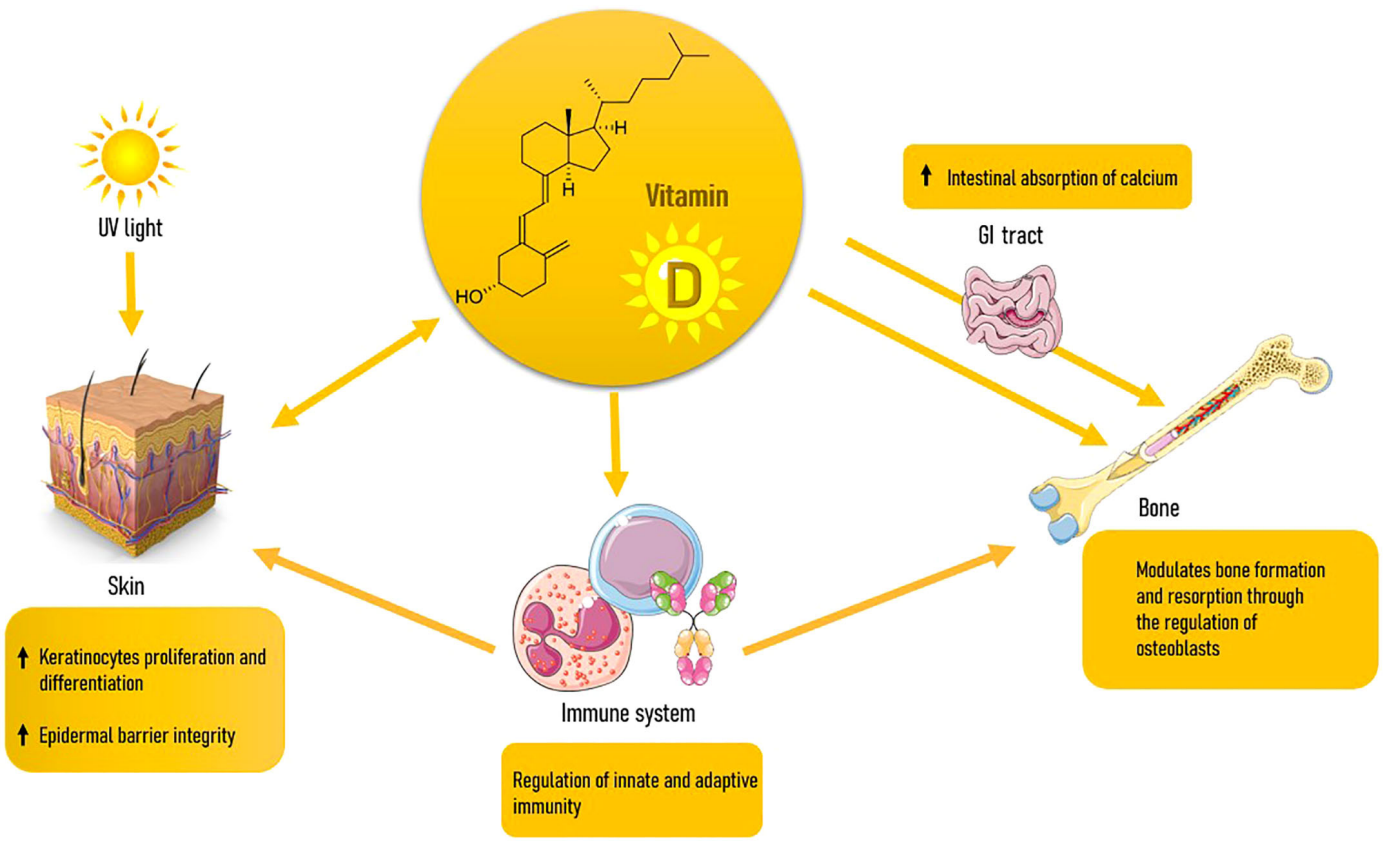
Interplays between Vitamin D, skin, bone and immune system.

Aging is marked by the continuous and progressive decline in organic functions and the increase of prevalence of chronic degenerative disease. OP and DP are expressions of this process at the bone and skin levels, respectively. Both conditions are associated with increased morbidity for elderly people, and this makes preventive interventions necessary. A first conclusion of our study is that since DP is a frequently observed condition by dermatologists, its presence might serve as a straightforward clinical indicator of bone frailty, encouraging healthcare providers to recognize and treat underlying OP. Furthermore, the two conditions share many risk factors, some not always editable such as corticosteroid use, others on which it is possible to intervene as VD deficiency. VD is involved in either disease with many mechanisms, among which immunomodulation (**Figure 2**). VD deficiency has been linked to OP because it inhibits the body’s capacity to absorb calcium and maintain optimal bone health. When it comes to skin, VD is involved in the formation, growth, and repair of skin cells. Both hypo and hypervitaminosis D appear to accelerate skin aging, with a U-shaped response curve to VD status. As a result, proper vitaminosis D appears to be vital in preventing age-related issues. DP is often seen in elderly individuals, particularly those with long-term sun exposure and a history of chronic sun damage. VD deficiency can be linked to DP, as it affects the quality and the composition of the skin. Although further research is needed to establish a definitive link between VD and DP, the existing evidence suggests its potential benefits in supporting skin health and mitigating the effects of this age-related condition. VD’s role in collagen production, epidermal barrier function, inflammation regulation, wound healing, and sun protection makes it a promising avenue for addressing DP.

More research with rigorous and reproducible evaluation is required to better understand the role of VD in the development of fragility syndromes as DP and OP, but since now it is advisable to maintain adequate levels of VD to prevent these conditions, as VD deficiency is a simply avoidable and curable condition with major health effects.

## Author contributions

FR and CDS contributed to ideation, drafting, and revising of the manuscript. FR, DS, CMT, AS, DAC, RA, ML and CDS, contributed to the literature search and drafting of the manuscript. AC contributed to ideation and revising of the manuscript. All authors have revised and accepted the final version of the manuscript.
